# Strategies to Reduce the Risk of Rectal Stump Leakage After Hartmann's Procedure: A Structured Narrative Review

**DOI:** 10.7759/cureus.108261

**Published:** 2026-05-04

**Authors:** Mohamed Alkashty, Ehab Kahka, Mafdi Mossaad, Waseem Hameed, Abanoub Saleeb, Ahmed Elshawadia, Mohamed Elgazawey

**Affiliations:** 1 General Surgery, Wexham Park Hospital, Slough, GBR

**Keywords:** colorectal surgery, endoscopic vacuum therapy, hartmann reversal, hartmann's procedure, pelvic sepsis, rectal stump leakage, rectal stump management

## Abstract

Rectal stump leakage (RSL) is a clinically significant complication following Hartmann's procedure, associated with substantial morbidity, prolonged hospital stay, and challenges during subsequent reversal surgery. This study presents a structured narrative review that provides a qualitative synthesis of the available evidence on mechanisms, risk factors, and strategies to reduce the risk of RSL. Given the heterogeneity of the literature, predominantly retrospective study designs, and inconsistent definitions of stump leakage, formal quantitative synthesis was not feasible. Available evidence suggests that RSL is a multifactorial process driven by impaired tissue healing, mechanical stress, technical factors related to stump construction, and an adverse pelvic environment. Reported associations include male sex, low body mass index, short rectal stump length, and prior pelvic radiotherapy, although the strength and consistency of these relationships vary across studies. Preventive strategies, including attention to stump length and placement, optimization of tissue perfusion, and reduction of intraluminal pressure through decompression techniques, may mitigate risk, but robust comparative data remain limited. Many proposed interventions are supported primarily by observational evidence or extrapolation from anastomotic leak literature. This review emphasizes a mechanism-based, risk-informed approach to prevention while clearly distinguishing between factors supported by quantitative signals and those that remain biologically plausible but unproven. Key gaps include the absence of validated risk prediction models, the lack of prospective Hartmann-specific data, and inconsistent outcome definitions. Future research should focus on prospective cohort studies, standardized reporting frameworks, and the development of clinically applicable risk stratification tools to guide intraoperative decision-making and improve outcomes following Hartmann's procedure.

## Introduction and background

Hartmann's procedure, involving resection of the rectosigmoid colon, closure of the rectal stump, and formation of an end colostomy, remains a commonly performed operation for complicated diverticulitis, colorectal cancer, and other emergent colorectal conditions. Despite its utility, rectal stump leakage (RSL) is a clinically significant postoperative complication, reported in approximately 11-17% of contemporary series, and is associated with pelvic sepsis, abscess formation, prolonged hospitalization, and increased mortality. In addition to its immediate consequences, RSL complicates subsequent reversal surgery, frequently resulting in permanent stoma formation and reduced quality of life. The pathogenesis of RSL is multifactorial, involving impaired tissue healing and perfusion, increased intraluminal pressure, technical factors related to stump construction, and an adverse pelvic environment [1-5].

The available evidence is limited by heterogeneous study designs, predominantly retrospective data, and inconsistent definitions of stump leakage, making it difficult to draw firm conclusions or establish standardized approaches to prevention. This review therefore provides a structured narrative synthesis of the current literature, focusing on factors associated with RSL and operative strategies that may reduce risk. It also highlights areas of uncertainty and ongoing controversy to support clinical decision-making and guide future research.

## Review

Literature search strategy

This study was conducted as a structured narrative review of the available literature. Relevant studies were identified through targeted searches of PubMed and Scopus, supplemented by screening the reference lists of key articles. Search terms included combinations of "Hartmann's procedure", "rectal stump", "rectal stump leakage", "stump leak", and "pelvic sepsis".

Studies were selected for their relevance to RSL following Hartmann's procedure, including those addressing incidence, risk factors, operative techniques, prevention, and management. Given the limited volume of procedure-specific evidence, selected adjacent colorectal literature was also considered where it provided mechanistic or clinically relevant insights. The included literature comprised predominantly retrospective and prospective cohort studies, as well as registry analyses, systematic reviews, technical reports, and narrative reviews.

Owing to heterogeneity in study design, patient populations, and definitions of RSL, a formal quantitative synthesis was not undertaken. Instead, findings were synthesized narratively using a structured framework based on the underlying mechanisms of stump failure, associated risk factors, and strategies for risk reduction. Preventive approaches were prioritized as the primary focus of the review, while diagnostic and management considerations were addressed as a secondary component.

As a narrative review, this study does not include predefined inclusion criteria, formal risk-of-bias assessment, or statistical pooling of data. These limitations are acknowledged, and the findings should be interpreted as a qualitative synthesis intended to provide a clinically oriented overview rather than definitive causal inference.

Review analysis

Risk Factors for RSL

Patient-related factors: Available evidence identifies several patient-related factors associated with an increased risk of RSL, including male sex, low body mass index (BMI), prior pelvic radiotherapy, chemotherapy exposure, and alcohol overconsumption [2,3,5]. These factors are likely to influence stump integrity by affecting tissue healing, local perfusion, and the pelvic operative environment.

Male sex is frequently reported and is often interpreted as a surrogate for increased technical difficulty, particularly in the context of a narrower and deeper pelvis, which may limit operative exposure and compromise optimal stump construction [2]. Low BMI and malnutrition are more consistently associated with impaired wound healing, reduced physiological reserve, and increased susceptibility to infection, all of which may predispose to stump failure [2]. Prior pelvic radiotherapy represents a particularly important risk factor, as radiation-induced fibrosis, microvascular injury, and chronic tissue ischemia can significantly impair tissue integrity and pelvic healing [3,5-7].

Tumor location below the peritoneal reflection and the requirement for low rectal transection have also been associated with an increased risk of leakage, likely reflecting both technical and biological challenges. Lower rectal stumps are more difficult to construct securely, may be subject to greater mechanical stress, and are less amenable to effective drainage within the pelvis [2]. In addition, severe intra-abdominal sepsis, including complicated diverticulitis and feculent peritonitis, creates an adverse inflammatory environment that further compromises healing and increases the risk of stump breakdown [3].

The roles of smoking and elevated BMI remain less clearly defined in the context of RSL. Although both factors are recognized contributors to impaired wound healing and anastomotic complications in colorectal surgery, their direct association with stump leakage following Hartmann's procedure has not been consistently demonstrated in procedure-specific studies. Similarly, while obesity may increase operative difficulty, prolong operative time, and adversely affect tissue perfusion, current evidence does not provide robust or consistent data linking high BMI to increased rates of stump leakage. In contrast, low BMI and malnutrition appear to be more consistently associated with impaired healing and leakage risk. These factors should therefore be considered as potentially contributory but unproven, highlighting an important gap in the existing literature.

Overall, these findings support a risk-informed approach to perioperative assessment, although the relative contribution of individual patient-related factors remains difficult to quantify due to limitations in the available evidence.

Operative and technical factors: Among operative variables, rectal stump length consistently emerges as an important determinant of outcome. Very short or ultra-low rectal stumps have been associated with higher rates of pelvic sepsis, increased technical difficulty at reversal, and a greater risk of stump blowout, particularly when transection is performed close to the pelvic floor [5,8,9]. This relationship is likely multifactorial, reflecting challenges in achieving secure closure, increased mechanical stress, and limited capacity for effective pelvic drainage. However, the association is not uniformly observed, and some studies have not demonstrated a significant predictive value of stump length for postoperative complications, particularly in the context of reversal surgery [10].

Additional operative factors may further influence stump integrity. Prior laparotomy and distorted pelvic anatomy can increase operative complexity, leading to more difficult dissection and a higher likelihood of tissue trauma [5,11]. Intraoperative variables such as prolonged operative time, increased blood loss, and technically demanding dissection have also been associated with a greater risk of postoperative complications, including stump leakage [12].

Vascular considerations are also relevant. High ligation of the inferior mesenteric artery may result in a relatively short and potentially compromised blood supply to the rectal stump, which may adversely affect healing and increase the risk of leakage. Furthermore, delayed reversal, particularly beyond six months, has been associated with progressive stump shrinkage and increased technical difficulty at reoperation. However, its direct impact on stump leakage remains less clearly defined [11].

Strategies to Reduce Risk

Preserve a reasonable stump length: Given the conflicting evidence regarding the impact of rectal stump length, a balanced and pragmatic interpretation is required. Several studies suggest that very short or ultra-low rectal stumps are associated with increased technical difficulty, higher rates of pelvic sepsis, and a greater risk of stump blowout, particularly when transection is performed close to the pelvic floor [5,8,9]. This may reflect challenges in achieving secure closure, increased mechanical stress, and limited capacity for effective pelvic drainage. However, this association is not consistently observed, and other studies have not demonstrated a clear relationship between stump length and postoperative outcomes, particularly following reversal [10].

Taken together, the available evidence does not support a defined threshold for optimal stump length. Nevertheless, from a practical standpoint, excessively short stumps may increase the risk of stump-related complications by compromising technical control during formation and limiting options for subsequent management. Accordingly, where oncological and anatomical considerations permit, avoiding an unnecessarily short rectal remnant represents a reasonable operative principle within a risk reduction strategy while recognizing that this recommendation is based on limited and heterogeneous evidence.

Attention to stump construction and fixation: The method of rectal stump closure has not been consistently shown to influence leakage risk. Available evidence does not demonstrate a clear or universal advantage of hand-sewn over stapled techniques in preventing stump-related complications [13,14]. Stapling may offer improved operative efficiency and facilitate technical standardization in appropriate settings [15]. Overall, outcomes appear to depend more on fundamental surgical principles, including meticulous tissue handling, secure closure, avoidance of tension, and preservation of adequate perfusion [16].

Adjunctive technical measures have been described to optimize stump management, although supporting evidence remains limited. Selective fixation of the rectal stump to the presacral fascia or abdominal wall has been proposed to reduce stump retraction and maintain accessibility [17,18]. While these strategies are primarily reported in the context of facilitating reversal, they may also indirectly benefit stump integrity by improving anatomical orientation and reducing the need for extensive dissection in a hostile pelvis. Similarly, simple measures such as marking the stump with a suture may assist identification and reduce tissue trauma during subsequent surgery [19].

Taken together, these findings suggest that attention to technical detail during stump construction is likely more important than the choice of closure method alone. However, the impact of fixation and adjunctive techniques on stump leakage remains uncertain, and their use should be considered pragmatic rather than evidence-based.

Decompression and drainage: Reducing intraluminal pressure is a plausible and potentially important strategy for minimizing the risk of RSL. One of the more consistent signals in the literature is the potential benefit of intraoperative rectal decompression using a Foley catheter [3]. The proposed mechanism is intuitive: decompression reduces intraluminal pressure, facilitates drainage of secretions, and thereby limits mechanical stress on the newly closed stump. Observational data suggest an association with reduced rates of stump-related complications, although the magnitude of effect remains uncertain and should be interpreted with caution [3].

Similar principles underpin the use of transanal tubes or stents, which have been explored more broadly in colorectal surgery. While these techniques may not eliminate leaks, they appear to reduce the severity of complications when leaks occur, likely by promoting controlled drainage and preventing pressure accumulation [20-22].

Overall, these findings support the concept that a freshly closed rectal stump, particularly in the setting of inflammation or sepsis, should not be exposed to unnecessary intraluminal pressure or impaired drainage. Decompression, whether achieved with a rectal Foley catheter, a transanal device, or an adjunctive drainage strategy, is a pragmatic consideration in selected high-risk cases, although robust comparative data remain limited.

Perfusion assessment: Although much of the available evidence on perfusion relates to colorectal anastomoses rather than specifically to rectal stump closure in Hartmann's procedure, the underlying principle remains directly relevant to stump integrity. Adequate perfusion is fundamental to tissue healing, and compromised vascular supply may predispose to stump failure and leakage. Near-infrared fluorescence angiography using indocyanine green has shown promise in assessing bowel perfusion and supporting intraoperative decision-making in colorectal surgery [23-27].

In the context of Hartmann's procedure, its role is largely extrapolated, as procedure-specific data are limited. Nevertheless, ensuring that the rectal stump is transected and closed on well-perfused, viable tissue represents a logical component of risk reduction. The principal limitation remains the absence of robust Hartmann-specific outcome data and standardized protocols; therefore, the clinical impact of perfusion assessment on stump leakage risk remains uncertain.

Minimally invasive approaches: Minimally invasive approaches, including laparoscopic and robotic Hartmann's procedures, where feasible and safe, have been associated with reduced wound morbidity, shorter length of stay, and potentially improved conditions for subsequent reversal [28-30]. These benefits are likely related to reduced tissue trauma, improved visualization, and lower abdominal wall disruption. In the context of RSL, such factors may indirectly promote healing by reducing surgical stress and preserving tissue integrity.

However, minimally invasive surgery is not inherently protective against stump-related complications, particularly where adequate control of sepsis and safe operative dissection are compromised. The choice of operative approach should therefore be guided primarily by the underlying pathology and intraoperative conditions, ensuring that principles of source control, tissue viability, and secure stump construction are not compromised in pursuit of a minimally invasive technique.

Tailor stump placement: Although much of the available evidence is derived from inflammatory bowel disease literature rather than Hartmann's procedure specifically, the underlying principles are transferable. Current data do not support a single universally superior position for the rectal stump. Subcutaneous placement may be advantageous in selected contaminated or high-risk settings, as stump failure is more likely to declare itself superficially, potentially limiting the severity of deep pelvic sepsis. Conversely, intraperitoneal placement may be preferable in other situations, taking into account patient comfort, wound-related considerations, and technical familiarity [31-33].

In the context of RSL, stump position may influence not only the likelihood of complications but also their clinical manifestation and ease of management. Accordingly, stump placement should be a deliberate and individualized decision, guided by the degree of contamination, tissue quality, patient body habitus, and anticipated plans for reversal, while recognizing that comparative evidence remains limited.

Postoperative Surveillance and Early Detection

Despite optimal operative technique, RSL cannot be completely prevented; therefore, the reduction of associated morbidity depends on timely recognition and intervention. Early detection relies on a structured, multimodal approach. Clinical assessment remains fundamental, and the use of validated scoring systems, such as the Colon Leakage Score (CLS) and the Dutch Leakage Score (DLS), may support earlier identification of patients at increased risk [34,35].

Serum biomarkers provide an additional layer of surveillance. C-reactive protein (CRP), particularly on postoperative day 3, has been identified as a sensitive early indicator of leakage, with suggested threshold values in the range of approximately 80-136 mg/L [36,37]. However, no single parameter should be interpreted in isolation, and trends in inflammatory markers should be considered alongside the clinical picture.

Imaging remains central to confirming the diagnosis. Computed tomography combined with rectal contrast enema (RCE-CT) has demonstrated high sensitivity and specificity for early detection and is increasingly regarded as the preferred modality when stump leakage is suspected [38,39].

In practice, the integration of clinical evaluation, laboratory markers, and targeted imaging offers the most reliable strategy for early detection. This approach facilitates timely intervention and may reduce the severity of pelvic sepsis and its associated complications [40].

Management of Established RSL

Principles of management: Once RSL is established, management is guided by the principles of surgical sepsis, including prompt source control, effective drainage, antimicrobial therapy, and physiological support. Early drainage is consistently emphasized, with image-guided techniques often sufficient in hemodynamically stable patients with localized pelvic collections. However, reoperation is required in more severe cases, particularly in the presence of diffuse peritonitis, ongoing sepsis, tissue necrosis, or failure of minimally invasive measures [1,41-44].

Open versus minimally invasive reintervention: When reoperation is indicated, the choice of approach should be determined by the need for definitive source control. Minimally invasive reintervention may be appropriate in selected patients and can reduce wound-related morbidity; however, open surgery remains the standard in the setting of severe sepsis, dense adhesions, or compromised tissue planes [44-47]. The priority should be adequate exposure, thorough washout, and secure control of the septic focus rather than adherence to a specific access technique.

Endoscopic and minimally invasive salvage: One of the most important developments in this field is the expanding role of endoscopic vacuum therapy (EVT/EVAC). Although much of the supporting evidence is extrapolated from the colorectal anastomotic leak literature, its use in RSL following Hartmann's procedure has been reported to yield encouraging results. EVT appears most beneficial in selected patients with localized leaks or contained cavities, particularly where repeated endoscopic access is feasible, and sepsis is controlled [48-56].

Other endoscopic modalities, including over-the-scope clips, tissue sealants, and transrectal drainage techniques, may have a role in carefully selected cases. These approaches should be considered adjuncts within a broader sepsis-control strategy rather than alternatives to adequate drainage in systemically unwell patients [57-59]. Recurrence of pelvic sepsis or abscess formation following apparently successful endoscopic salvage remains a recognized limitation, underscoring the need for careful patient selection and close follow-up.

Hartmann's Reversal and the Importance of Stump Preservation

Although the primary focus of this review is the prevention of RSL at the index operation, evidence from Hartmann's reversal provides relevant indirect insights into factors influencing stump integrity and long-term outcomes [9-11,17,30,45,60-63]. Delayed reversal has been associated with stump shrinkage, fibrosis, adhesion formation, and increased technical difficulty at reoperation [11,45,61-63]. Some studies suggest that, where clinically appropriate, reversal within approximately 3-6 months may be advantageous; however, this timing should be individualized based on patient condition and disease factors [61-63].

These observations reinforce the importance of constructing a rectal stump that is not only secure in the acute setting but also accessible for future reconstruction. Preoperative imaging before reversal is often valuable, particularly when stump length, residual collections, or anatomical uncertainty may influence operative planning [9,11,64]. In addition, technical considerations at the index procedure, including preservation of an adequate stump length, appropriate fixation, and intraoperative marking, may facilitate subsequent surgery and reduce operative complexity.

Practical Surgical Bundle for Risk Reduction

Based on the available evidence, a pragmatic approach to risk reduction following Hartmann's procedure may include the following considerations: (1) early identification of patients at increased risk, including those with severe intra-abdominal sepsis, low BMI or malnutrition, prior pelvic radiotherapy, alcohol misuse, and anticipated low rectal transection [2,3,5-7]; (2) preservation of an adequate rectal stump length where disease extent and operative safety permit, avoiding unnecessarily short stumps [5,8-10]; (3) construction of the stump on well-perfused, tension-free tissue, with meticulous handling and adherence to sound surgical technique [16,23-27]; (4) consideration of stump fixation or marking to reduce retraction and facilitate identification at reversal, where appropriate [17-19,39,40]; (5) selective use of decompression strategies, such as rectal Foley catheter or transanal drainage, particularly in higher-risk settings [3,20-22]; (6) deliberate choice of stump placement (intraperitoneal versus subcutaneous), guided by contamination, tissue quality, and patient-related factors [31-33]; (7) vigilant postoperative surveillance, with a low threshold for imaging, particularly CT with rectal contrast, when recovery deviates from the expected course [38-40]; (8) early control of pelvic sepsis through timely drainage, appropriate antimicrobial therapy, and escalation to operative intervention when required [1,41-44]; (9) selective use of endoscopic salvage techniques, including endoscopic vacuum therapy, in appropriately selected and clinically stable patients [48-56]; and (10) thoughtful planning of reversal, avoiding unnecessary delay where clinically appropriate, and ensuring adequate preoperative assessment [10,11,61-64]. These elements should be interpreted as a pragmatic framework rather than a prescriptive protocol, reflecting the limitations and heterogeneity of the available evidence.

Discussion

RSL is best understood as a multifactorial complication rather than the consequence of a single technical failure. It reflects the interaction between patient-related factors, disease severity, operative technique, and postoperative course. This complexity likely explains the absence of a single consistently effective preventive manoeuvre and supports a pragmatic, risk-adapted approach to management.

Among operative variables, rectal stump length remains an area of ongoing debate. While very short or ultra-low stumps have been associated with increased pelvic complications and technical difficulty during reversal [5,8,9], this relationship has not been consistently demonstrated across studies [10]. In practice, stump length must be considered alongside oncological requirements, pelvic conditions, and anticipated reconstruction. By contrast, certain patient-related factors, particularly prior pelvic radiotherapy and low BMI, appear more consistently associated with impaired healing and increased leakage risk [2,3,5-7].

Reduction of intraluminal pressure represents a plausible mechanism for risk reduction. The reported association between rectal decompression, including intraoperative Foley catheter use and transanal drainage, and lower rates of stump-related complications suggests a potential role for these strategies in selected patients [3,20-22]. However, the available data are predominantly observational, and the magnitude of benefit remains uncertain.

Taken together, these findings support a structured, risk-informed approach. Patients with recognized risk factors, such as severe intra-abdominal sepsis, low BMI, prior radiotherapy, or technically demanding pelvic dissection, may benefit from targeted intraoperative considerations, including careful stump construction, attention to perfusion, and selective use of adjuncts such as decompression or tailored stump positioning [2,3,5,11,12,16,31-33]. Postoperative surveillance also remains critical, with the integration of clinical assessment, biomarkers, and imaging enabling earlier detection and intervention [34-40].

Management of established leakage continues to rely on timely sepsis control, with increasing use of minimally invasive and endoscopic techniques in selected cases, including endoscopic vacuum therapy [48-56]. Early diagnosis is central to the success of these approaches. While minimally invasive strategies for both index surgery and reintervention may reduce morbidity in appropriate patients, their use should not compromise definitive source control [28-30,44-47].

The current evidence base remains limited. Much of the available literature is retrospective, heterogeneous, and not specific to Hartmann's procedure, with frequent reliance on extrapolation from related colorectal settings [23-27,31-33]. There are a lack of standardized definitions of RSL, limited reporting of operative variables, and the absence of validated risk prediction models. In addition, the independent contribution of factors such as smoking, obesity, and abdominal wall characteristics remains poorly defined. As a narrative review, this study is also subject to inherent selection bias and does not provide a quantitative synthesis.

Future research should focus on prospective, procedure-specific datasets with standardized outcome definitions, enabling more reliable risk stratification and evaluation of preventive strategies.

In summary, prevention of RSL is best approached through a combination of careful risk assessment, sound operative technique, selective use of adjuncts, and vigilant postoperative monitoring, rather than reliance on any single intervention.

## Conclusions

RSL following Hartmann's procedure is a clinically significant and likely under-recognized complication that requires a structured, proactive approach to risk reduction. The available evidence suggests that risk is multifactorial, with no single intervention sufficient in isolation; rather, a combination of careful patient assessment, sound operative technique, including avoidance of an unnecessarily short rectal stump and construction on well-perfused, tension-free tissue, and selective use of adjunctive measures such as decompression may contribute to improved outcomes in selected cases. Vigilant postoperative surveillance remains essential for early detection and timely intervention. However, the current evidence base is limited by heterogeneity, reliance on retrospective data, and a lack of procedure-specific studies, and further work is needed to establish standardized definitions, develop prospective Hartmann-specific datasets, and evaluate pragmatic strategies applicable to routine emergency colorectal practice.

## References

[1] Johnston S, de Lacavalerie P (2020). Management of rectal stump leak following emergency Hartmann's procedure. J Coloproctol.

[2] Huang X, Xiao Z, Huang Z (2023). Rectal stump leakage: a neglected complication after Hartmann's procedure for colorectal cancer. Surgery.

[3] Secher J, Balachandran R, Iversen LH (2023). Incidence and risk factors of blowout within 90 days after a primary Hartmann's procedure: a retrospective cohort study. Langenbecks Arch Surg.

[4] Sverrisson I, Nikberg M, Chabok A, Smedh K (2018). Low risk of intra-abdominal infections in rectal cancer patients treated with Hartmann's procedure: a report from a national registry. Int J Colorectal Dis.

[5] Ritter AS, Dumm N, Deisenhofer JM, Franz C, Al-Saeedi M, Büchler MW, Schneider M (2024). Risk factors for rectal stump leakage after discontinuity resection: stump length matters most. Dis Colon Rectum.

[6] Jonker FH, Tanis PJ, Coene PP, van der Harst E (2015). Impact of neoadjuvant radiotherapy on complications after Hartmann procedure for rectal cancer. Dis Colon Rectum.

[7] Wang G, Wang W, Jin H (2022). Effect of abdominal circumference on the irradiated bowel volume in pelvic radiotherapy for rectal cancer patients: implications for the radiotherapy-related intestinal toxicity. Front Oncol.

[8] Westerduin E, Musters GD, van Geloven AA, Westerterp M, van der Harst E, Bemelman WA, Tanis PJ (2017). Low Hartmann's procedure or intersphincteric proctectomy for distal rectal cancer: a retrospective comparative cohort study. Int J Colorectal Dis.

[9] Reali C, Landerholm K, George B, Jones O (2022). Hartmann's reversal: controversies of a challenging operation. Minim Invasive Surg.

[10] Patteet E, Van Hoof S, Hendrickx T, Van den Broeck S, Hubens G, Komen N (2022). Is length of the rectal stump predictive for postoperative outcome in Hartmann's reversal surgery? A multicenter experience of 105 consecutive cases. Int J Colorectal Dis.

[11] Rajcoomar MS, Mewa Kinoo S, Naidoo R, Singh B (2017). The challenges of the Hartmann's rectal stump reversal: a clinical audit and review of the literature. Int Surg.

[12] Teerawiwatchai C, Thongin A, Prasitvaraku K (2025). Risk factors for anastomotic leakage after rectal surgery in locally advanced rectal cancer. J Health Sci Med Res.

[13] Vignali A, Gozzini L, Gasparini G, Calef R, Rosati R, Elmore U (2023). Impact of powered circular stapler on anastomotic leak after anastomosis to the rectum: a propensity score matched study. Int J Colorectal Dis.

[14] Kshirsagar VV, Mp H (2024). A comparative study of hand-sewn and stapled anastomosis in gastrointestinal surgeries. Cureus.

[15] Munawar F, AlKhalifa S, AlTraiki Z (2025). Meta analysis of stapled vs hand-sewn anastomosis in gastrointestinal surgery. J Surg Res.

[16] Jones DW, Garrett KA (2014). Anastomotic technique-does it make a difference?. Semin Colon Rectal Surg.

[17] Fukada A, Ogino T, Fujimoto Y (2025). A proactive technique for reversal of Hartmann's procedure: lifting the rectal stump to the abdominal wall. Tech Coloproctol.

[18] Velitchkov Velitchkov, Vassilev Vassilev, Belokonsky Belokonsky, Losanoff Losanoff, Kjossev Kjossev (1999). A novel method to avoid pelvic adhesions following Hartmann's procedure. Colorectal Dis.

[19] Madura JA, Fiore AC (1983). Reanastomosis of a Hartmann rectal pouch: a simplified procedure. Am J Surg.

[20] Yang CS, Choi GS, Park JS, Park SY, Kim HJ, Choi JI, Han KS (2016). Rectal tube drainage reduces major anastomotic leakage after minimally invasive rectal cancer surgery. Colorectal Dis.

[21] Jones M, Moran B, Heald RJ, Bunni J (2024). Can the Heald anal stent help to reduce anastomotic or rectal stump leak in elective and emergency colorectal surgery? A single-center experience. Ann Coloproctol.

[22] Choy KT, Yang TW, Heriot A, Warrier SK, Kong JC (2021). Does rectal tube/transanal stent placement after an anterior resection for rectal cancer reduce anastomotic leak? A systematic review and meta-analysis. Int J Colorectal Dis.

[23] Lee KH (2026). Fluorescence-guided surgery in colorectal cancer: current evidence, quantitative advances, and future perspectives. Ann Coloproctol.

[24] Charbonneau J, Papillon-Dion É, Brière R (2025). Fluorescence angiography with indocyanine green for low anterior resection in patients with rectal cancer: a prospective before and after study. Tech Coloproctol.

[25] Iwamoto M, Ueda K, Kawamura J (2022). A narrative review of the usefulness of indocyanine green fluorescence angiography for perfusion assessment in colorectal surgery. Cancers (Basel).

[26] Faber RA, Meijer RP, Droogh DH (2024). Indocyanine green near-infrared fluorescence bowel perfusion assessment to prevent anastomotic leakage in minimally invasive colorectal surgery (AVOID): a multicentre, randomised, controlled, phase 3 trial. Lancet Gastroenterol Hepatol.

[27] Belloni E, Muttillo EM, Di Saverio S, Gasparrini M, Brescia A, Nigri G (2022). The role of indocyanine green fluorescence in rectal cancer robotic surgery: a narrative review. Cancers (Basel).

[28] Bradea C, Tarcoveanu E, Munteanu V, Lupascu CD, Andriesi-Rusu FD, Ciobanu DG, Vasilescu AM (2023). Laparoscopic Hartmann procedure-a surgery that still saves lives. Life (Basel).

[29] Shao YC, Shen MY, Chen WT (2023). Laparoscopic Hartmann's procedure. Mastering Endo-Laparoscopic and Thoracoscopic Surgery.

[30] Lee KY, Lee J, Oh ST (2023). Postoperative effects of laparoscopic Hartmann reversal: a multicenter propensity score matched study. PLoS One.

[31] Gallo G, Kotze PG, Spinelli A (2018). Surgery in ulcerative colitis: when? How?. Best Pract Res Clin Gastroenterol.

[32] Poritz LS (2006). Consequences of the retained rectum in inflammatory bowel disease. Semin Colon Rectal Surg.

[33] Vitello D, McGee MF (2022). Treatment of severe and fulminant inflammatory bowel disease colitis. Semin Colon Rectal Surg.

[34] van Beekum CJ, Beckmann C, Semaan A (2022). Predictors of morbidity and mortality after colorectal surgery in patients with cirrhotic liver disease-a retrospective analysis of 54 cases at a tertiary care center. Front Med (Lausanne).

[35] Bertocchi E, Barugola G, Ceccaroni M, Guerriero M, Rossini R, Gentile I, Ruffo G (2022). Laparoscopic colorectal resection for deep infiltrating endometriosis: can we reliably predict anastomotic leakage and major postoperative complications in the early postoperative period?. Surg Endosc.

[36] Lyu Z, Wu D, Cai G (2018). Use of C response protein in predicting postoperative anastomotic leakage in patients with rectal cancer [Article in Chinese]. Zhonghua Wei Chang Wai Ke Za Zhi.

[37] Lee WT, Varghese P, Gaunt A (2026). Post-operative C-reactive protein as a predictor of anastomotic leak following robotic colorectal surgery. World J Surg.

[38] Moreno-Lopez N, Mvouama S, Bourredjem A (2023). CT scan for early diagnosis of anastomotic leak after colorectal surgery: is rectal contrast useful?. Tech Coloproctol.

[39] Kauv P, Benadjaoud S, Curis E, Boulay-Coletta I, Loriau J, Zins M (2015). Anastomotic leakage after colorectal surgery: diagnostic accuracy of CT. Eur Radiol.

[40] Yung HC, Daroch AK, Parikh R, Mathur DV, Kafexhiu IK, Goodman E (2024). Diagnostic modalities for early detection of anastomotic leak after colorectal surgery. J Surg Res.

[41] Martins BC, Marques CF, Nahas CS (2012). A novel approach for the treatment of pelvic abscess: transrectal endoscopic drainage facilitated by transanal endoscopic microsurgery access. Surg Endosc.

[42] Schein M, Kopelman D, Nitecki S, Hashmonai M (1993). Management of the leaking rectal stump after Hartmann's procedure. Am J Surg.

[43] Bosscha K, van Vroonhoven TJ, van der Werken C (1999). Surgical management of severe secondary peritonitis. Br J Surg.

[44] Shaw D, Beaty JS, Thorson AG (2015). Reoperative surgery for diverticular disease and its complications. Semin Colon Rectal Surg.

[45] Garber A, Hyman N, Osler T (2014). Complications of Hartmann takedown in a decade of preferred primary anastomosis. Am J Surg.

[46] Kwak JM, Kim SH, Son DN (2011). The role of laparoscopic approach for anastomotic leakage after minimally invasive surgery for colorectal cancer. J Laparoendosc Adv Surg Tech A.

[47] Ma R, Wu G, Chilla S, Solis-Pazmino P, Barnajian M, Nasseri Y (2025). Minimally invasive converted to open versus upfront open surgeries for rectal cancer: a retrospective cohort study. Surg Endosc.

[48] Shalaby M, Emile S, Elfeki H, Sakr A, Wexner SD, Sileri P (2019). Systematic review of endoluminal vacuum-assisted therapy as salvage treatment for rectal anastomotic leakage. BJS Open.

[49] Kühn F, Zimmermann J, Beger N (2021). Endoscopic vacuum therapy for treatment of rectal stump leakage. Surg Endosc.

[50] Chopra SS, Mrak K, Hünerbein M (2009). The effect of endoscopic treatment on healing of anastomotic leaks after anterior resection of rectal cancer. Surgery.

[51] Chorti A, Stavrou G, Stelmach V, Tsaousi G, Michalopoulos A, Papavramidis TS, Kotzampassi K (2020). Endoscopic repair of anastomotic leakage after low anterior resection for rectal cancer: a systematic review. Asian J Endosc Surg.

[52] Clifford RE, Fowler H, Govindarajah N, Vimalachandran D, Sutton PA (2019). Early anastomotic complications in colorectal surgery: a systematic review of techniques for endoscopic salvage. Surg Endosc.

[53] Kuehn F, Janisch F, Schwandner F, Alsfasser G, Schiffmann L, Gock M, Klar E (2016). Endoscopic vacuum therapy in colorectal surgery. J Gastrointest Surg.

[54] Saavedra JE, Carrillo CA, Valbuena GL (2022). Outpatient closure in a late colo-cutaneous postoperative anastomotic leak managed with EVAC in Bucaramanga, Colombia. Case report. Int J Surg Case Rep.

[55] Popivanov GI, Mutafchiyski VM, Cirocchi R, Chipeva SD, Vasilev VV, Kjossev KT, Tabakov MS (2020). Endoluminal negative pressure therapy in colorectal anastomotic leaks. Colorectal Dis.

[56] Riss S, Stift A, Kienbacher C (2010). Recurrent abscess after primary successful endo-sponge treatment of anastomotic leakage following rectal surgery. World J Gastroenterol.

[57] Arezzo A, Verra M, Reddavid R, Cravero F, Bonino MA, Morino M (2012). Efficacy of the over-the-scope clip (OTSC) for treatment of colorectal postsurgical leaks and fistulas. Surg Endosc.

[58] Chiari D, LA Raja C, Mangiavillano B, Veronesi P, Platto M, Zuliani W (2022). Multimodal treatment of colorectal postsurgical leaks: long-term results of the over-the-scope clip (OTSC) application. Minerva Surg.

[59] Cwaliński J, Hermann J, Paszkowski J, Banasiewicz T (2023). Dehiscence of colorectal anastomosis treated with noninvasive procedures. Wideochir Inne Tech Maloinwazyjne.

[60] Christou N, Rivaille T, Maulat C (2020). Identification of risk factors for morbidity and mortality after Hartmann's reversal surgery - a retrospective study from two French centers. Sci Rep.

[61] Popazu C, Voicu D, Firescu D, Grigore I, Toma A, Derihaci RP (2025). Optimal timing of colostomy reversal following Hartmann's procedure: a retrospective analysis of postoperative outcomes. Diseases.

[62] Roe AM, Prabhu S, Ali A, Brown C, Brodribb AJ (1991). Reversal of Hartmann's procedure: timing and operative technique. Br J Surg.

[63] Vaghiri S, Stylianidi MC, Engelmann L (2025). Reversal of Hartmann's procedure: the impact of timing - a single-tertiary-center experience. Surg Pract Sci.

[64] Friedman S, Ricci ZJ, Stein MW, Wolf EL, Ekinci T, Mazzariol FS, Kobi M (2019). Colostomy on CT and fluoroscopy: what the radiologist needs to know. Clin Imaging.

